# Perception and experience of clinicians and caregivers in treating childhood severe pneumonia and hypoxemia using bubble continuous positive airway pressure in Ethiopian tertiary and general hospitals

**DOI:** 10.1371/journal.pone.0275952

**Published:** 2022-10-31

**Authors:** Meseret Gebre, Md. Fakhar Uddin, Trevor Duke, Kassa Haile, Md. Tanveer Faruk, Mehnaz Kamal, Md. Farhad Kabir, Abebe Genetu, Rahel Argaw Kebede, Asrat Demtse, Abate Yeshidinber Weldetsadik, Abayneh Girma Demisse, Bitseat W. Haile, Alemseged Abdissa, Teferi Elfu, Biruk Tesfaye, Taye Tolera Balcha, Muluye Shemeles, Tahmeed Ahmed, John D. Clemens, Mohammod Jobayer Chisti

**Affiliations:** 1 Armauer Hansen Research Institute, Addis Ababa, Ethiopia; 2 Nutrition and Clinical Services Division (NCSD), icddr,b, Dhaka, Bangladesh; 3 Centre for International Child Health, Royal Children’s Hospital, The University of Melbourne, Melbourne, Australia; 4 Tikur Anbessa Specialized Hospital, Addis Ababa, Ethiopia; 5 St. Paul’s Hospital Millennium Medical College, Addis Ababa, Ethiopia; 6 Yekatit 12 Hospital Medical College, Addis Ababa, Ethiopia; 7 Butajira General Hospital, Addis Ababa, Ethiopia; 8 Bishoftu General Hospital, Addis Ababa, Ethiopia; University of Notre Dame Australia, AUSTRALIA

## Abstract

**Background:**

In low and middle-income countries (LMICs), severe pneumonia with hypoxemia is the leading cause of child deaths, even with the provision of WHO-recommended antibiotic therapy, oxygen therapy and other supportive care. Previous studies found positive outcomes from the use of bubble continuous positive airway pressure (bCPAP) for treating these children compared to the standard oxygen therapy. Due to lack of data on the perceptions and experiences of hospital health care workers and caregivers of children on the feasibility and acceptability of bCPAP in treating children with severe pneumonia and hypoxemia in real-life settings, we examined these issues in tertiary and general hospitals in Ethiopia.

**Methods:**

As part of a three-stages clinical trial, this qualitative study was conducted in two tertiary (stage I) and two general (stage II) hospitals from September 2019 to July 2020. During stages I and II, we have consecutively enrolled children with severe pneumonia and hypoxemia and put them on bCPAP to examine its feasibility and acceptability by clinicians and parents. A total of 89 children were enrolled (49 from two tertiary and 40 from two general hospitals). Then qualitative data were collected through 75 repeated in-depth interviews by social-science experts with purposively selected 30 hospital health workers and 15 parents of 12 children who received bCPAP oxygen therapy in the hospitals. Interview data were supplemented by 6 observations in the hospitals. Data were analyzed using a thematic approach.

**Results:**

Identified structural and functional challenges for the introduction of bCPAP in treating childhood severe pneumonia and hypoxemia in the study hospitals include: inadequate number of pulse oximeters; unavailability of nasal prongs with age-specific size; inadequate and non-functioning oxygen flow meters, concentrator, and cylinders; disruption in power-supply; and inadequate number of staff. The opportunities in introducing bCPAP oxygen therapy included the availability of a dedicated corner for the study patients situated in front of nurse’s station, required medicines and satisfactory level of clinicians’ knowledge and skills for treating severe pneumonia patients. Additionally, the identified operational challenges were occasional lack of bubbling in the water-filled plastic bottle, lack of stand for holding the water-filled plastic bottle, and delayed shifting of oxygen source from an oxygen concentrator to a cylinder, particularly during electricity disruption. Participants (clinicians and parents) expressed their satisfaction as bCPAP oxygen therapy was found to be simple to handle, children had ease of breathing and recovered fast without major ill effects.

**Conclusion:**

Our study identified some important structural, functional, and operational challenges that need to be addressed before implementation of bCPAP oxygen therapy especially in frontline general hospitals with limited resources. In spite of these observed challenges, the clinicians and caregivers were highly satisfied with the overall performance of bCPAP oxygen therapy.

## Background

Pneumonia is a leading cause of death in children under five years old and accounts for 15% of all deaths in this age category [1]. In 2018, an estimated 5.3 million children died globally, and majority of those deaths occurred in developing countries including Sub-Saharan Africa [2]. In Ethiopia, acute respiratory infection, particularly pneumonia, is the leading cause of morbidity and mortality in under five children, and accounts for 18% of all under-five deaths [2]. Hypoxaemia (arterial oxygen saturation <90% in room air) [3], is one of the main risk factors for death due to pneumonia among children [4]. Hypoxaemia has been observed in 13–41% of children requiring hospitalization for World Health Organisation (WHO) classified severe pneumonia [5, 6]. Despite the provision of WHO-recommended standard oxygen therapy, antibiotics and supportive care, the case fatality rate for children with severe pneumonia and hypoxaemia is still high in health facilities of resource limited settings [7, 8]. In developed countries, in addition to antibiotics and other supportive care, the use of high-flow oxygen therapy, humidification of the high-flow mixture of air and oxygen via nasal oxygen cannula and continuous positive airway pressure via mechanical ventilator are possible options for treating children with moderate or severe respiratory distress [9]; however, this range of tools are not available for treatment of most children with severe pneumonia and hypoxemia in low and middle income countries (LMICs).

Continuous positive airway pressure (CPAP) oxygen therapy has been widely used for children with moderate to severe respiratory distress in intensive care units (ICU) in developed countries. CPAP can be delivered in a number of ways: mechanical ventilator via tracheal tube; mechanical ventilator via face mask/nasal prongs; flow driver/bCPAP via face mask or nasal prongs. The most common method to deliver CPAP is via a mechanical ventilator, which is not available in most health facilities in developing countries. The use of mechanical ventilators to deliver CPAP generally requires greater expertise and advanced training of healthcare providers [10, 11]. These technical challenges often limit the availability of CPAP to health care facilities, even in higher level clinical care settings. However, bubble CPAP (bCPAP) can be delivered using simple and low-cost material, as shown elsewhere [12]. bCPAP with inexpensive nasal prongs that can be connected with an oxygen concentrator for treating children with severe pneumonia and hypoxemia. This method generates positive-end-expiratory pressure by connecting the expiratory limb of a breathing circuit to a tube, which is submerged in water. The distance that the distal end of the expiratory tube is under water is equivalent to the pressure (in cm H2O) that will be generated in the upper airway if an adequate seal has been made in the patient interface and bubbles appear in the water bottle [12]. Positive pressure delivered to those with lower airway disease (i.e. pneumonia) makes breathing more comfortable, reduces respiratory rate, improves airway clearance, and consequently improves ventilation [13, 14].

bCPAP has been used successfully in neonatal respiratory distress in developed countries since the 1970s, and has been promoted for its simplicity, low cost, and potential applicability for neonatal care in LMICs settings since early 2000’s [15, 16]. Moreover, a previous efficacy trial conducted at the International Centre for Diarrhoeal Disease Research, Bangladesh (icddr,b) in the paediatric intensive care unit of its Dhaka Hospital found positive outcomes of bCPAP compared to the standard low flow oxygen therapy for treating children with severe pneumonia and hypoxemia beyond the neonatal age [13]. A systematic review which included ten studies and an efficacy trial revealed the favourable outcome of the use of bCPAP oxygen therapy for treating children with severe pneumonia and hypoxemia [14]. It is also important to note that after the end of the Bangladeshi trial, bCPAP has been used routinely to support nearly 1,000 children with severe pneumonia and hypoxemia over a period of more than four years at the Dhaka hospital of icddr,b with outcomes consistent with the findings of the efficacy trial [17]. Even though the beneficial effect of bCPAP has been shown in different studies, it is important to assess its feasibility and acceptability in low resourced real-life settings for subsequent scale-up where low-flow oxygen therapy is still the standard of care for hospitalized children with severe pneumonia and hypoxemia.

Studies done in Australia and Canada described different oxygen delivery methods as stressful to parents/guardians, as they create physical barriers between guardians and children [18, 19]. Communicating treatment plans and supporting caregivers are essential to healthcare, which is a challenge in understaffed hospitals [20]. Frequently, caregivers receive inadequate health information about a child’s illness, management, and prognosis [21], leading to caregivers having unanswered questions about their child’s condition [22] and possible medico-legal actions [23]. One descriptive study, which was done in Malawi, reported some challenges relating to implementation of bCPAP among infants with severe pneumonia. Initially caregivers received inadequate, inconsistent, and sporadic information about bCPAP from health workers; caregivers felt anxious and fearful—when their infants were on bCPAP due to limited parent–child interaction and the constraints of prescribed visiting hours [24]. Key perceived opportunities included hospital health workers’ ability to clarify caregivers’ questions and concerns and involvement of family, friends, and parents/guardians in the care of patients [25]. This study was conducted in a hospital and identified the gaps in the information and psychological support that mothers of infants on bCPAP receive in the hospital. However, perceptions and experiences of clinicians and caregivers of the children regarding clinical use, maintenance and acceptability of bCPAP oxygen therapy have not yet been explored in-depth. Therefore, we aimed to understand the feasibility and acceptability of bCPAP oxygen therapy for treating children with severe pneumonia and hypoxemia in real life hospital settings from the perceptions and experiences of health workers and caregivers of children in Ethiopia. The output of this study will be used to guide researchers, policy makers and hospital administrators to make important decisions on the design and conduct of a subsequent multi-center implementation trial for scaling-up the bCPAP oxygen therapy to frontline hospital settings in Ethiopia.

### Ethiopian context: Tertiary and general hospitals

In Ethiopia, medical college hospitals and general hospitals are considered as tertiary and district hospitals respectively. These hospitals usually provide care of patients in paediatric wards that include children with severe pneumonia. To treat severe pneumonia, general hospitals can typically provide WHO recommended drugs and supplementary oxygen by oxygen cylinder or concentrator but the availability of pulse oximeter is limited. Most general hospitals do not have additional respiratory support such as mechanical ventilation or CPAP available to children who are failing to improve on low flow (LF) oxygen therapy. As a result, children with severe pneumonia who are failing with LF may need to be referred to tertiary hospitals, which may not be always possible due to financial constraints or lack of bed in tertiary hospitals. We explored the feasibility and acceptability of introducing bCPAP in both tertiary and general hospitals as a prelude to a large implementation trial.

## Methods

### Study design

As part of a three-stage clinical trial, this descriptive phenomenological qualitative study [26, 27] was conducted in two tertiary (stage I) and two general (stage II) hospitals of Ethiopia from September 2019 to July 2020. For this trial, bCPAP was introduced in the above-mentioned hospitals for treating children aged 1–59 months with severe pneumonia. This trial was followed by a multicenter implementation trial (stage III) taking into consideration the qualitative findings revealed in stage I and stage II. This qualitative study was implemented through a participatory approach by actively engaging all stakeholders related to the bCPAP oxygen therapy including consumers or beneficiaries (caregivers/ parents or guardians) and hospital clinicians (physicians and nurses). The aim of this integrated approach was to fill the gaps in the available information, to provide different perspectives on complex, contextual, and multi-dimensional phenomena.

### Study settings

We conducted this study in two tertiary hospitals, *Yekatit 12 Hospital Medical College* and *St*. *Paul’s Milleneum Medical College* hospital and two general hospitals, *Butajira* hospital and *Bishoftu* hospital. Both tertiary teaching hospitals were located in Addis Ababa, Ethiopia. Each month, an average of 80–100 and 30–40 children with severe pneumonia were admitted into the tertiary hospitals and general hospitals respectively. During the study period the oxygen delivery method in all hospitals was LF oxygen therapy (WHO recommended) with occasional use of bCPAP in critical patients. Children with severe pneumonia are placed on IV antibiotics and other supportive care including maintenance fluid. Children with an acute or chronic respiratory failure, defined as insufficient oxygenation, insufficient alveolar ventilation, or both were put on mechanical ventilators depending on its availability.

General hospitals had limited facilities for care of these children (where there were less manpower and medical equipment) compared to tertiary hospitals. Both general hospitals had mechanical ventilators, but rarely use them for pediatric patients because most of the time mechanical ventilators were occupied by adult patients. Critical patients are referred to the hospitals in Addis Ababa or elsewhere as needed.

### Recruitment of participants and data collection

A total of 49 and 40 children with severe pneumonia and hypoxemia aged 1–59 months were provided bCPAP in tertiary (n = 27 in *Yekatit*, and n = 22 in *St*. *Paul’s* hospitals) and general hospitals (n = 20 in *Butajira*, and n = 20 in *Bishoftu* hospitals) respectively. For this qualitative study, we purposively [26, 28] identified 28 participants (parents, n = 12 and clinicians, n = 16) from tertiary hospitals and 17 participants (parents, n = 3 and clinicians, n = 14) from general hospitals to conduct in-depth interviews. These participants were closely involved in the care of children with severe pneumonia and hypoxemia receiving bCPAP. We selected different parents/guardians based on their involvement in continued patient care, their different levels of education and age groups of their children. We purposively selected qualified hospital physicians and nurses who were involved in the study and were working in the pediatrics department for at least 6 months.

We interviewed clinicians of the hospitals on two occasions (first occasion—within the first week and second occasion—within the last three weeks during implementation of bCPAP in the hospitals) and caregivers of the children on one occasion after completion of the bCPAP oxygen therapy course, holding a total of 15 in-depth interview with caregivers-family members and 60 in-depth interviews with clinicians. Moreover, data collected from interviews were supplemented by data from 6 observations (n = 2 in general and n = 4 in tertiary hospitals) at a single approach for one patient. Each observation was made from 8a:m in the morning to 5p:m in the evening. Social science experts actively observed the behavior and interaction of each child and his/her parent/guardian while the children were receiving bCPAP. Interviewers and observers had a social science academic background and were experienced in collecting qualitative data locally. They were trained by a qualified anthropologist. They conducted interviews in the hospitals at a place and time convenient for the participants. The average duration of an interview was 47 min. Interviews were conducted using flexible semi-structured data collection guides and an audio-recorder. The language we used for taking interview was Amharic, but translator to other local language was used whenever needed. Interview guides were prepared based on the study objectives and were pilot-tested. We continued data collection until the point of data saturation was achieved.

### Data analysis

Qualitative interviews were transcribed to verbatim and observation notes were written up into detailed notes just after the accomplishment of fieldwork. After completion of translation of each transcript and observation note by the local interviewers, two researchers coded each transcript and observation notes, compared results, and resolved any discrepancies to ensure trustworthiness of the coding process. We used a thematic coding approach (according to our study objectives) and a framework emerged from interviews and observational data and the thematic coding enriched our narrative analysis. Data were analyzed manually, using both thematic and narrative approaches [26, 29]. We followed a sequence of inter-related steps [29] that included reading, coding, re-reading, displaying data, reducing, and interpreting textual data using emic approach [30] after which the main themes were finalized. However, the complex issues were presented in some verbatim forms.

## Ethical approval

The study was approved by the Ethical Review Committee of icddr,b, AAERC (AHRI / ALERT Ethics Review Committee), NRERC (National Research Ethics Review Committee) and EFDA (Ethiopia Food and Drug Administration). We obtained written informed consent from participants for all interviews, recordings and observations.

## Results

### Patients’ and participants’ characteristics

The main characteristics of purposively selected 12 children (who received bubble CPAP oxygen therapy) for this qualitative study are summarized in Table 1. Of the 12 children, 6 were from tertiary and 6 were from general hospitals. Three parents/guardians of children from general hospitals were illiterate and four were housewives—engaged primarily in subsistence agriculture. Most children (n = 9) were less than two years. In addition, age of the clinicians (n = 30) who participated in this study was less than 40 years and sixteen clinicians had more than five years work experience in the hospitals, particularly in pediatric ward. They had medical education from different nursing institutions and medical college hospitals in Ethiopia. Twenty-three clinicians were familiar in treating patients with severe pneumonia and hypoxemia using oxygen from their academic life.

**Table 1:** Characteristics of study patients and participants.

Background characteristics	Number of patients (n = 12) /participants	
	Tertiary hospitals	General hospitals
Sex of patients		
Male	3	3
Female	3	3
Age of patients		
0–11 months	3	2
12–23 months	1	3
24–59 months	2	1
Relationship of parents/guardians with patients		
Mother	5	5
Father	1	1
Age of parents/guardians		
20–29 years	3	1
30–39 years	3	4
40–49 years	-	1
Education of parents/guardians		
Illiterate	-	3
Primary level	2	2
Secondary level	1	1
Higher secondary level	1	-
Diploma	2	-
Occupation of parents/guardians		
Service	4	-
Business	1	1
Farmer	-	1
Housewife	1	4

In this section, we presented findings under the key major themes which included: challenges and opportunities related to structural and functional condition of the hospitals; operational challenges related to clinical use and maintenance of bCPAP oxygen therapy intervention; and programmatic and behavioral acceptability of o bCPAP oxygen therapy. Key findings of the study are summarized in Table 2.

**Table 2:** Operational challenges related to the clinical use and maintenance of bCPAP.

Parameters	Stage I–tertiary hospitals	Stage II–general hospitals
Hospitals	St. Paul’s & Yekatit	Bishoftu & Butajira
Lack of bubbling in the water filled plastic bottle (reported by doctors and nurses)	Especially in mouth breathers, timely action taken	Especially in mouth breathers, timely action taken
Nasal secretion and dryness of nose (reported by doctors and nurses)	timely action taken	timely action taken
Lack of stand for holding water filled plastic bottle	Complaints both from clinicians and caregivers	Complaints both from clinicians and caregivers
Shifting of bCPAP from O2 concentrators to cylinder	Timely action taken	Delay in two cases at night
Anecdotal experiences of doctors and nurses	Simple to handle, no adverse events, rapid recovery, reduced bed occupancy and HAI	Simple to handle, no adverse events, rapid recovery, reduced bed occupancy and HAI
Overall experiences of caregivers	Initial anxiety (some), eventual happiness (all) with the positive outcome (easy breathing, smiling)	Initial anxiety (some), eventual happiness (all) with the positive outcome (easy breathing sleeping, opening eyes, smiling)

### Challenges related to structural and functional condition of the hospitals

Initially, we identified some structural and functional challenges in both tertiary and general hospitals, which included inadequate number of pulse oximeters, less availability of nasal prongs with age specific size, and non-functioning oxygen flow meters. Overcoming these challenges was essential for the successful implementation of bCPAP intervention.

#### Pulse oximeters

Clinicians expressed that initially, an inadequate number of pulse oximeters hampered the follow-up activities related to bCPAP oxygen therapy in tertiary and general hospitals. For instance, there was only one portable pulse oximeter in the pediatric ward in a tertiary hospital, and in another tertiary hospital, there was a non-functioning pulse oximeter due to the lack of proper maintenance. Therefore, sometimes clinicians borrowed pulse oximeters from the emergency room or triage center. The lack of pulse oximeters was solved by supplying two pulse oximeters to each study hospital by the study team. Clinicians thought that apart from making the availability of pulse oximeters in the hospitals, keeping the maintenance of the existing devices is also an important issue.

#### Oxygen flow meters

Clinicians in tertiary and general hospitals reported that some oxygen flow meters did not work for a long time because of low-quality. Therefore, they faced difficulty to estimate the true flow of oxygen while children were receiving bCPAP using an oxygen cylinder. Clinicians suggested that purchasing good quality equipment could be the solution.

*“We have oxygen flow meters*, *some of them are not working properly*. *Some of them don’t have gauge i*.*e*. *if you want to give two-liter oxygen per minute for a patient*, *the flow meter gauge doesn’t work and does not show the amount of liter and sometimes we provide oxygen blindly*, *i*.*e*. *we didn’t know the volume of oxygen that the patient received*.*” ID-01*, *Nurse*, *29 years*, Yekatit hospital

#### Nasal prongs with age-specific size

Clinicians mentioned that at the beginning of introduction of bCPAP in the tertiary and general hospitals, they had difficulty in fitting nasal prongs to nostrils of some small children (aged less than 6 months) receiving bCPAP. They also often faced a similar challenge in case of WHO standard low flow oxygen therapy as they used similar nasal interface. So, they used micropore (adhesive tape) to fix the nasal prong into the nostrils, but sometimes the nasal prong was displaced from the child’s nostrils. They identified the problem when they observed no bubble in the water-filled plastic bottle during receiving the bCPAP treatment. Therefore, study physicians suggested ensuring the availability of nasal prongs with different age-specific sizes in the pediatric ward during implementation of bCPAP oxygen therapy. Later, age-specific nasal prongs were supplied by the study team and they were found suitable for the study children. Although the study team provided different sized nasal prongs to the hospitals for the study purpose, the clinicians added it would not be a permanent solution in the future until they are made available to all the tertiary and general hospitals.

We also found some structural and functional challenges, particularly in general hospitals, such as inadequate and non-functioning oxygen concentrators. In addition, disruption in refilling oxygen cylinders, lack of power generator back-up and inadequate number of staff were also observed. We explain below the key challenges identified in general hospitals.

#### Oxygen concentrators

Hospital clinicians mentioned that available concentrators in the general hospitals were very old, and unable to create enough oxygen flow to produce adequate bubbling. However, the study team supplied two brand new concentrators to each of the general hospitals for this study purpose. One clinician stated that this was not a permanent solution and the concentrators were not adequate. Clinicians reported about the shortage of oxygen concentrators particularly at the time of high patient flow in the pediatric ward, which might disrupt the implementation of bCPAP in the general hospitals. During such situation, clinicians were obliged to borrow oxygen concentrators from other departments (e.g. ICU).

*“There were two oxygen concentrators in the pediatric ward provided by the study team*. *Sometimes two of them were occupied at the same time by the study patients*. *So*, *when another child with severe pneumonia came to the pediatric ward having no additional concentrator for bCPAP*, *I think two concentrators for bCPAP treatment is not adequate*. *The solution may be to shift newly enrolled patients to oxygen concentrator occupied by old study patient (receiving bCPAP) and old study patients could be shifted to oxygen cylinder*.*”* IDI-01, Physician, 33 years, Butajira hospital

One clinician from a general hospital was skeptical about the suitability of bCPAP treatment in other remote general hospitals as she thought shortage of oxygen concentrators would be a big challenge.

#### Oxygen cylinders and power generator back-up

Clinicians from general hospitals mentioned that generally the hospital had a shortage of oxygen cylinder supply as oxygen cylinders have to be delivered and refilled from Addis Ababa, but for the case of the bCPAP study, they used mainly oxygen concentrators, which facilitated oxygen therapy.

Participants (clinicians and caregivers) reported that there was a repeated interruption of bCPAP treatment in the general hospitals due to the failure of electric power supply, as oxygen concentrators run by electric power. The duration of electric power interruption was different; sometimes it took a few minutes and sometimes longer. The chance of failure of both the power sources (electricity and the generator) was rare, but occurred a few times. For instance, clinicians of Butajira hospital experienced a power cut problem in the pediatric ward for two hours and the automatic power generator was not working that night. In this situation, the patient had to be switched from oxygen concentrator to oxygen cylinder. Two caregivers reported delayed oxygen support for their children during the power failure problem due to mal-functioning of available oxygen cylinders. Therefore, clinicians felt that adequate number of oxygen cylinders and continuous electricity supply are very crucial for strengthening the quality of oxygen therapy via bCPAP, particularly in general hospitals.

However, power failure was not a problem in tertiary hospitals. The automatic generator was connected to the system in case of electric power failure, so it took only a fraction of a minute to secure the electricity.

#### Staffing

Clinicians from general hospitals mentioned that patients on bCPAP treatment needed to be followed up consistently to complete data according to the study protocol, but the follow-ups required by the study were difficult for the study clinicians particularly during night shifts and weekends. The shortage of staff became worse when a physician was sent for a training course.

*“There are three working shifts in the hospital—morning shift 8*:*00 am-1*:*30 pm*, *afternoon shift 1*:*30 pm-5*:*30 pm and night shift 5*:*30 pm- 7*:*30 am*. *So at least one of the nurses is expected to be present in each shift which was very challenging for us*. *The night shift has overtime payment*, *so every nurse in the ward (11 nurses in total) wants to get the benefit from it*. *So*, *the night shift was equally distributed for these 11 nurses by roster*. *So*, *some days there were shifts where the nurse for the study patients was not scheduled*.*”* IDI-04, Nurse, 31 years, Butajira hospital

Other clinicians, who were not involved in this study and did not receive training on bCPAP, were not cooperative in providing the necessary support to the study clinicians during follow-ups of the patients with bCPAP in absence of study clinicians in the ward. Therefore, study clinicians felt that before the implementation of the new bCPAP in other general hospitals, all staff in the pediatric department need to be trained.

Hospital clinicians related to study had to perform their study related activities in addition to their routine hospital work, which affected follow-ups of the patients on bCPAP. The pediatric ward in the general hospitals was very crowded and sometimes created difficulty for the clinicians to perform required study related follow-ups. One clinician emphasized the need for full-time research staff with a strong commitment to the successful implementation of bCPAP. Another clinician felt that the supervision of patients receiving bCPAP would be done more effectively if the clinicians have focused attention in managing such patients. However, a nurse thought that if bCPAP becomes part of the standard of care in the hospital in treating all severe pneumonia patients, all staff in the pediatric ward will be routinely trained to implement the treatment effectively, thus it would not be an additional workload for the staff. Although the shortage of human resources and workload were the challenges to follow-up of patients on bCPAP in general hospitals, dedication and self-commitment of clinicians (including pediatricians) made it possible to follow the patients effectively.

One clinician from *Butajira* hospital indicated that the hospital might lose staff trained on bCPAP due to the high rate of staff turnover, mainly due to the fact that everyone looks for better options and on average the maximum duration of staying in a hospital or ward is 1–2 years.

*“There is a trend of the hospital called “staff rotation” and every year each staff in the hospital rotates from one department to another department*. *This means trained nurses and physicians on bCPAP may not stay in the pediatric department in the coming year*, *so this might create a gap in the implementation of bCPAP treatment*. *So*, *before the implementation of bCPAP treatment as a routine care*, *the hospital human resource should consider the staff rotation schedule*.*”* IDI-04, Physician, 34 years, Bishoftu hospital

### Opportunities related to structural and functional condition of the hospitals

There were some opportunities in tertiary and general hospitals related to structural and functional conditions such as an available bCPAP treatment corner (dedicated space or room), and a satisfactory level of clinicians’ knowledge and skills in treating severe pneumonia patients. For instance, clinicians of tertiary hospital perceived that adopting bCPAP would not be challenging in tertiary hospitals as clinicians have been using and working with CPAP (both machine driven and bubble CPAP) for treating newborns with respiratory distress for a long time. Only a short training would be sufficient for nurses and physicians on bCPAP treatment for children with severe pneumonia. However, in hospitals where bCPAP treatment is totally new, there might be minor initial challenges. Moreover, there was an added advantage of tertiary hospitals in implementing bCPAP, as they had adequate oxygen supply with availability of oxygen concentrators and cylinders.

### Operational challenges related to bCPAP oxygen therapy

We explored a range of operational challenges, including occasional lack of bubbling in the water-filled plastic bottle (especially in mouth breathers), nasal secretions, dryness of the nostrils, and lack of a stand for holding the water-filled plastic bottle to protect the bottle from fall and splash. Below is the detailed description of these challenges and their potential solutions.

#### Lack of bubbling in the water-filled plastic bottle

In tertiary and general hospitals, clinicians recognized that there was occasional lack of bubbling in the water-filled plastic bottle for the first few cases especially among mouth breathers until clinicians became familiar with the whole process. Therefore, clinicians checked all the possible causes such as a blockage in the circuit, leaking in the oxygen source/circuit, or cessation of oxygen in the cylinder to solve the problem related to lack of bubbles inside the water bottle and fixed the problem.

*“I observed interruption of bubble production during the treatment of my child using bCPAP*. *While the child was in my lap*, *I think the tube became loose in the nose of my daughter during my movement and this might be the reason for the absence of bubbling*. *In terms of solving the problem*, *they [health workers] told me to report to them [health worker] if I see interruption of bubble production*. *So*, *I followed their advice and they came to us and solved the problem accordingly (e*.*g*. *fixed the bCPAP)*.*”* IDI-02, Mother, 25 years, St.Paul hospital

#### Nasal secretions and dryness of the nostrils

Clinicians mentioned that during administration of bCPAP oxygen therapy, the patient sometimes developed nasal secretions followed by nasal blockage and dryness of the nostrils. They reported that initially in some cases, the oxygen saturation of the patient was not improving because of the nasal blockage.

*“I have seen nasal secretion causing nasal blockage in a child receiving bCPAP treatment*. *The child had a common cold and the nasal cavity was blocked by mucus*. *Probably the oxygen cannot overcome the nasal blockage to reach the lung of the child*.*”* IDI-03, Nurse, 31 years, Yekatit hospital

The above-mentioned challenges were easily solved by clinicians using saline with cotton for cleaning the nasal passages. Then routine follow-ups were made until the children showed improvement in their illness.

#### Lack of a stand for holding the water-filled plastic bottle

Clinicians and caregivers mentioned that during the implementation of bCPAP, placement and handling of the water-filled plastic bottle was difficult to control due to the lack of a bedside holder for the bottle in both tertiary and general hospitals. Clinicians learnt from the training that the water-filled plastic bottle should be placed below or at the height of the level of the patient to have adequate bubble and pressure and to avoid resistance. However, in practice, it was difficult to find a convenient place beside the bed to hold the water-filled plastic bottle. Clinicians thought that the treatment might be affected by the position of the water bottle.

Caregivers faced difficulty in handling both the bCPAP water bottle and their children simultaneously. A physician opined that sometimes caregivers were moving the water bottle to different positions as they required moving their children in their lap and during this process water inside the bottle poured out through the hole on the top of the bottle. Therefore, the depth of water inside the bottle decreased and affected the pressure coming out of the bottle. So, clinicians used to advise caregivers to seek help to solve such incidents like bottle fall and water splash.

*“The water-filled plastic bottle fell down and water splashed several times when I moved with my child*. *Then I called the nurses to fix it again*. *In addition to routine check-ups*, *nurses also checked the bottle when they entered into the room for any other reason such as medication*. *I think the bottle needs some adjustment*, *like giving or creating some holder that can protect the bottle from fall*.*”* IDI-01, Father, 28 years, St. Paul hospital

#### Other operational challenges

Clinicians reported that children above one year were more hyperactive than children below one year. Older children were more non-cooperative and often tried to remove the nasal prong from their nostrils (but this observation is also common in children receiving low flow oxygen). Sometimes it was difficult for clinicians and caregivers to handle such children. Therefore, clinicians gave their full attention to monitoring these children, particularly just after the introduction of bCPAP to check and re-fix the nasal prong into the child’s nasal canals. Moreover, in tertiary hospitals, at the initial stage, the noise of the oxygen concentrator made the children and their caregivers nervous. Therefore, caregivers had suggested having a potential solution for minimizing the noise.

*“I heard when nurses were talking among themselves about the noise of the concentrator*. *They said when the machine works for a long time it made noise*, *thus the machine required rest*. *To provide rest for oxygen concentrator*, *they switched the oxygen source to the cylinder*.*”* IDI-02, Mother, 36 years, Yekatit

### Programmatic and behavioral acceptability related to bCPAP oxygen therapy

Clinicians were satisfied with bCPAP treatment as it was very simple to handle, and has the capacity to accelerate patient recovery, had no adverse events, reduced bed occupancy and hospital-acquired infection (HAI), and decreased direct and indirect costs for the patients and hospitals. Furthermore, caregivers of the children were pleased with bCPAP, although, some of them initially felt anxious. Eventually, all caregivers were happy with positive outcomes (improvement of breathing and cough better sleeping) which were beyond their expectations. We illustrate below the programmatic and behavioral acceptability of bCPAP based on the real-life experiences of clinicians and caregivers of the children.

#### Experiences of clinicians (doctors and nurses)

*Rapid recovery*. In the study hospitals, clinicians expressed their contentment while treating patients using bCPAP as it required less time to improve the oxygen saturation of the patient. Physicians observed signs of improvement (oxygen saturation level) in some cases within the first hour and most of the cases within 48 hours. They also added that usually, the patient took 72 hours to maintain adequate oxygen saturation with low flow oxygen treatment. In addition, clinicians reported that most patients receiving bCPAP required less hospital stay (2–3 days) which is beneficial both for the patients and the hospital. They also explained that patients on low flow oxygen therapy usually needed to stay in the hospital for at least five to seven days. Clinicians reported some children recovered faster (in 12 hours) and some took longer (100 hours) depending on the severity of illness, child’s lung pathology, and other complication and/or co-morbidities of the patient.

*“To my observation*, *in the last twenty days about twenty children with severe pneumonia had received the bCPAP oxygen therapy and among them*, *eight children have been discharged with a better outcome or improved condition and others are recovering fast—though yet not discharged*.*”* IDI-03, Nurse, 31 years, Yekatit hospital

Therefore, clinicians thought that if the treatment will be available in other tertiary and general hospitals of Ethiopia, mortality rates among children with severe pneumonia would be decreased.

*Reduced bed occupancy and risk of Hospital Acquired Infection (HAI)*. Clinicians mentioned that the quick recovery of patients using bCPAP saved clinicians’ time, reduced patients’ bed occupancy, and patients’ load in the hospitals. These created opportunities for other patients with severe pneumonia to take treatment in the hospitals. They also thought that patients’ shorter hospital stay due to rapid recovery can also reduce the risk of developing hospital-acquired infections among the patients. Therefore, clinicians strongly felt the necessity of implementing bCPAP treatment for children with severe pneumonia in other tertiary and general hospitals of Ethiopia.

*“I have seen that children receiving bCPAP oxygen therapy are recovering faster than the children with low-flow oxygen therapy*. *Patient with bCPAP treatment requires maximum two to three days for complete recovery of severe pneumonia*, *but patients on low flow oxygen therapy required longer stay in the hospital for recovery–even some patients required to stay one week or more*. *So*, *I believe that bCPAP has positive impact on the hospital as it can reduce bed occupancy because of its capacity for quick recovery—where another severe pneumonia patient can use the bed for getting their emergency treatment support*. *Therefore*, *I strongly feel the necessity of initiating bCPAP for the treatment of severe pneumonia patients in other tertiary hospitals of Ethiopia”*. IDI-01, Physician, 32 years, St. Paul hospital

*Reduction of cost*. Clinicians indicated that the short hospital stay because of early recovery of illness using bCPAP reduced the cost of patients and hospitals. They thought that a short hospital stay can help in further reduction of oxygen consumption. They experienced that longer duration of low flow oxygen therapy requires more oxygen per patient, which is reduced by the use of bCPAP. Thus, lower hospital oxygen costs is an additional benefit of using bCPAP.

Moreover, bCPAP requires low-cost medical equipment to construct the device. Clinicians stated that it does not need extra items to operate; it only requires a WHO nasal prong, and a plastic bottle and water, which are not costly.

Clinicians felt that using an oxygen concentrator for bCPAP treatment reduced the oxygen cost of hospitals and patients as it can generate oxygen from air/environment and does not require refilling oxygen like oxygen cylinder. Furthermore, clinicians reported that the use of an oxygen concentrator for bCPAP instead of oxygen cylinder is an opportunity, particularly for the remote general hospitals, as they are situated far away from the oxygen production center. Refilling oxygen cylinders from outside of the hospitals has a high transportation cost and is also time-consuming.

*Simple to handle*. Clinicians reported that bCPAP oxygen therapy is very simple and easy to handle with existing medical equipment, and does not need to use any new complex equipment or technology and sophisticated skill of health workers. They also mentioned that it is easy for them to follow-up the patients as they use water-filled plastic bottles which are transparent and clear to see the bubbling inside the bottle. They felt that any hospital which has been practicing existing low flow oxygen therapy can adopt the bCPAP oxygen therapy easily.

*Minor ill events*. Clinicians observed minor nasal irritation, but no pneumothorax among those who received bCPAP. They mentioned that adverse events were averted due to successful accomplishment of required routine follow-ups. The follow-ups also had a great contribution to identify potential signs of impending complications and to take actions before the development of a complication.

Clinicians reported that bCPAP oxygen therapy is very safe for the patient if bCPAP is used with routine follow up. One physician expressed that the generation of higher flow oxygen and CPAP was very useful to improve respiratory fatigue within a short time, which saved the lives of children with severe pneumonia.

#### Experiences of caregivers

Initially, caregivers were anxious and doubtful about the positive outcome of bCPAP treatment, until they began to observe improvement of breathing difficulty of their children. They were concerned about the child’s crying with flushed face, dry nose, and the noise of oxygen concentrator. These events led caregivers to perceive that bCPAP might be harmful to their children. Caregivers expressed that the initial few minutes were very difficult for them (fear and worry) to accept the new treatment despite having the mental support from the clinicians and close relatives. This occurred as their children felt mild discomfort at the beginning of fixing the nasal prong into their nostrils, but within one hour of the onset of bCPAP treatment, they started to respond in a good way (e.g., children stopped crying and felt comfortable). Clinicians reported that this kind of discomfort is not only seen in bCPAP but was also observed among children receiving low flow oxygen as WHO nasal prong was used in both interventions.

Four caregivers mentioned that initially, although they consented, they became nervous as they were not aware of the possible responses to bCPAP treatment just after the introduction of bCPAP. Later the clinicians explained to them how bCPAP oxygen therapy works, which made caregivers happy and relieved their anxiety. Therefore, the clinicians understood the importance of providing routine mental support/ clarifications to any queries on bCPAP oxygen therapy. They added that this clarification and mental support may help future increase in accepting the intervention.

*“I was worried when I saw the oxygen flow was high and going to the child’s nostrils*. *I thought all the oxygen reach inside her stomach and might be harmful or painful for my child*. *I didn’t know that the amount of gas she inhaled would come out during exhalation*. *Then I called the nurses and almost decided to remove the bCPAP from my child*. *However*, *the clinicians understood and listened to my worries with empathy*, *and counseled me and then I understood it is not a problem at all*, *rather the high flow of oxygen might help the child to take breath easily*. *Eventually*, *within an hour both my child and I became comfortable*.*”* IDI-03, Mother, 36 years, Bishoftu hospital

After few hours of bCPAP treatment, all caregivers were happy, relaxed, and satisfied when they observed all signs and symptoms (e.g. fever, cough, vomiting, sneezing) were slowly disappearing and the condition of their children improved (easier breathing, sleeping, spontaneous eye opening, smiling, eating, playing), which was beyond their expectations. So, caregivers were grateful to clinicians as they offered them with such a novel treatment.

*“My child was critically ill*, *I was anxious last Thursday and Friday when my child was not awake*. *Even the physician in the evening shift told us that we should be ready for every outcome (even for death)*, *but later we had observed his condition was improving in a good way with the bCPAP oxygen therapy*. *I have no words to explain—how much I have been satisfied with this treatment*.*”* IDI-02, Caregiver, 36 years, Yekatit hospital

*“Most of the signs and symptoms that were severe at the time of admission like fever*, *cough*, *and respiratory difficulty*, *improved over time significantly*. *The child is now playful and can move both his hands and legs which were not seen before the treatment*. *My child has taken both treatments (new and existing one)*, *so I witnessed how the new treatment was effective*.*”* IDI-01, Father, 42 years, Bishoftu hospital

## Discussion

Initially, we identified several important challenges related to the structural and functional capacity of the study hospitals for successful implementation of the bCPAP oxygen therapy which included: shortage of pulse oximeters and flow meters; unavailability of nasal prongs with age-specific size; non-functional oxygen flow meters; inadequate and non-functioning oxygen concentrators and oxygen cylinders; disruption in refilling oxygen cylinders and power generator back-up; and inadequacy of staff. Most of these challenges were found in the general hospitals.

Interviewees expressed the concerns that there is a shortage of medical equipment in both the tertiary and general hospitals such as pulse oximeter, oxygen flow meters, and nasal prongs with age-specific size. Our study findings are consistent with a previous study conducted in Ethiopia, which reported that shortages of medical supplies and equipment were a widespread problem in health facilities at all levels [24]. However, our study revealed that shortage of medical equipment, particularly oxygen concentrators and cylinders, was encountered more in general hospitals than in the tertiary hospitals. Typically, in tertiary hospitals, equipment like oxygen concentrators, pulse oximeter, and nasal prongs were available. However, the number of available oxygen concentrators was not adequate to put every child admitted with severe pneumonia due to a high caseload in these settings. In such situations, clinicians were obliged to use sources of oxygen other than oxygen concentrators such as cylinders or central oxygen pipes. At times there was some resistance from senior clinicians on the logic behind putting every child with severe pneumonia and hypoxemia on bCPAP as they believed that it should be reserved for those who are not responding to standard WHO low flow oxygen therapy. In addition, we found that some medical equipment such as pulse oximeters, oxygen flow meters, and oxygen concentrators were available in the hospitals but some of them were non-functional. For instance, our study revealed that the available oxygen concentrators were not able to provide an adequate flow of oxygen to create optimal bubbling. Some also had a maximum flow rate of oxygen at 5 liter per minute, which made it impossible to titrate and increase the flow of oxygen in cases where children needed a higher positive end-expiratory pressure (PEEP), whereby oxygen flow needed to be increased in parallel to the depth of water level up to 10 cm requiring oxygen flow of 10 liters per minute. Therefore, the functionality of these medical equipment is another important issue that needed taking into consideration for the successful implementation of the bCPAP intervention in the hospitals. This study findings are also consistent with the previous study that reported medical equipment was often broken, or inappropriate for use [24]. Therefore, we suggest that ensuring the availability, functionality, good quality and proper maintenances of the existing medical equipment of the hospitals is very important for the successful implementation of the bCPAP treatment.

Trained, experienced and skilled health workforce shortages were found to be a critical obstacle to implementing bCPAP intervention, particularly in the general hospitals. This was not a major problem in the tertiary hospitals, where we observed an adequate number of pediatricians, general practitioners, pediatric residents and pediatric nurse specialists. These findings are also consistent with previous studies that reported that the number of neonatologists, pediatricians, and neonatal nurse specialists in the country were few and concentrated in tertiary centers [24, 25]. However, our study findings emphasize the importance of the availability of physician supervision for the effective implementation of bCPAP in general hospitals. Moreover, it is also important that the nurses working in the pediatric ward need to be involved for successful implementation of bCPAP because of the fact that trained study physicians sometimes may not be available in the pediatric ward to ensure required follow-up for patients receiving bCPAP. Overall, children on bCPAP therapy needed regular and consistent follow-up to make sure the circuit was not blocked or leaking, the nasal prong was well fitted and the depth of water level was maintained to assure adequate pressure was generated. This requires assistance from general nurses and other mid-level health workers in pediatric wards. Strengthening the capacity of such staff in remote facilities will require provision of training on bCPAP oxygen therapy.

Our study revealed that mothers were well informed by clinicians about bCPAP treatment and its potential outcome on recovery of child’s pneumonia, but they were anxious initially due to irritability of their children during placement of bCPAP. Clinicians recognized that caregivers may need additional information and support to reduce their fear. However, a previous study conducted in Malawi reported that most mothers knew that bCPAP provides extra oxygen to infants in severe respiratory distress, but were unable to tell the name of the machine, and feared bCPAP treatment outcome [27]. As such, it is important to reinforce and repeat information and allow time for parents to ask questions to clarify their understanding so that they meaningfully participate in the care of their children [31]. It is the responsibility of health workers to give adequate information, when possible, before any treatment to reduce stress [32]. To this end, in our study, study clinicians provided adequate information to the caregivers about the bCPAP and obtained written consent before introducing bCPAP to their children. This suggests a need for healthcare providers to explain the treatment plan comprehensively, which may include functions of the bCPAP oxygen therapy, benefits, and potential complications, as this will prepare caregivers psychologically and help them adapt better to the situation.

### Limitation and strength of the study

Qualitative data collection was limited in two tertiary and two general hospitals of Ethiopia. However, our study findings can be transferable to similar hospitals of Ethiopia.

On the other hand, there were a number of strengths of this study. We collected data using multiple qualitative data-collection methods including in-depth interviews and observations, and gathered perspectives of participants with diverse characteristics including physicians, nurse and patients’ parents/guardians. This helped us explore in-depth experiences and insights of participants and perform triangulation of findings from different angles as much as possible.

## Conclusion

Overall, our study showed that bCPAP can be feasible and acceptable to implement both in tertiary and general hospitals after addressing identified potential challenges that include assurance of routine clinical monitoring supervised by physicians, availability of necessary equipment with their good maintenance, retention of adequately trained staff, and good clinical setup as these are important for the successful implementation of bCPAP at a larger scale in low resource settings. Special consideration and attention should be given to hard to reach areas where power supply and shortage of medical equipment (e.g., oxygen concentrators, cylinder) would be a big challenge. In addition, our study findings have emphasized the importance of providing relevant information and mental support continuously for parents/guardians by clinicians throughout the course of the intervention in order to reduce their fears and worries.

## List of abbreviations

AAERC: ALERT AHRI Ethics Review Committee
bCPAP: bubble continuous positive airway pressure
EFDA: Ethiopia Food and Drug Administration
HAI: hospital acquired infections
ICU: intensive care units
IDI: in-depth interview
icddr,b: International Centre for Diarrhoeal Disease Research, Bangladesh
LMICs: low and middle-income countries
LF: low flow
NCSD: Nutrition and Clinical Services Division
NRERC: National Research Ethics Review Committee
PEEP: positive end-expiratory pressure
SIDA: Swedish International Development Cooperation Agency
WHO: World Health Organisation
